# Mortality and Predictors of In-Hospital Death Among Mechanically Ventilated Adults With Gram-Negative Pneumonia

**DOI:** 10.7759/cureus.113518

**Published:** 2026-07-28

**Authors:** Nasar Nasrullah Khan, Saeeda Shahid, Asim Hussain Rafila, Hubba Farid Jabbar, Spogmaye Haroon Ur Rashid, Muhammad Shahzad, Waqas Ahmed

**Affiliations:** 1 Medicine, Nottingham University Hospitals, Nottingham, GBR; 2 Pediatric Emergency Medicine, Burjeel Royal Hospital, Al Ain, ARE; 3 General Medicine, Ali Medical Centre, Islamabad, PAK; 4 Neurology, Oslo Universitets Sykehus, Oslo, NOR; 5 Geriatrics, Royal Liverpool University Hospital, Liverpool, GBR; 6 Emergency Department, Pakistan Ordnance Factory (POF) Hospital, Islamabad, PAK; 7 Cardiac Anesthesia, Hameed Latif Hospital, Lahore, PAK; 8 Medical Emergency and ICU, Mayo Hospital, Lahore, PAK

**Keywords:** critically ill patients, in-hospital mortality, multidrug-resistant, organism isolated, predictors

## Abstract

Background

Critically ill adults with gram-negative pneumonia who require invasive mechanical ventilation have a high risk of adverse outcomes and in-hospital mortality.

Objective

The objective of this study is to determine the in-hospital mortality rate and identify predictors of mortality among intubated and mechanically ventilated adult patients with gram-negative pneumonia.

Methods

This retrospective cohort study included 178 adult patients with microbiologically confirmed gram-negative pneumonia requiring endotracheal intubation and invasive mechanical ventilation. Demographic characteristics, comorbidities, severity scores, microbiological profile, antimicrobial resistance patterns, laboratory parameters, and clinical outcomes were reviewed. A total of 178 gram-negative isolates were identified.

Results

The overall in-hospital mortality rate was 76/178 (42.7%). The most frequently isolated pathogens were *Acinetobacter baumannii* in 58/178 isolates (32.6%), *Klebsiella pneumoniae* in 47/178 isolates (26.4%), and *Pseudomonas aeruginosa* in 35/178 isolates (19.7%). Multidrug-resistant organisms were identified in 93/178 patients (52.2%), while carbapenem resistance was observed in 67/178 isolates (37.6%). Non-survivors were significantly older than survivors and had higher APACHE II and Sequential Organ Failure Assessment (SOFA) scores. Septic shock was present in 48/76 non-survivors (63.2%) versus 24/102 survivors (23.5%), multidrug-resistant infection in 51/76 non-survivors (67.1%) versus 42/102 survivors (41.2%), and inappropriate empirical antibiotic therapy in 36/76 non-survivors (47.4%) versus 23/102 survivors (22.5%).

Conclusion

In-hospital mortality among mechanically ventilated patients with gram-negative pneumonia was high. Advanced age, greater illness severity, septic shock, multidrug-resistant infection, inappropriate empirical antibiotic therapy, hypoalbuminemia, and elevated lactate levels were significant predictors of mortality.

## Introduction

Pneumonia continues to be one of the major diseases contributing to morbidity and mortality in the world, especially if the patient is critical and needs to be admitted to the intensive care unit (ICU) [1]. Severe pneumonia may result in acute hypoxemic respiratory failure, which often requires endotracheal intubation and mechanical ventilation. While there have been significant advances in critical care management, mortality rates are still high among mechanically ventilated patients with pneumonia (25-50% depending on patient factors, pathogens, severity and healthcare setting) [2]. Gram-negative bacteria are one of the most frequent causes of serious hospital-acquired pneumonia (HAP), ventilator-associated pneumonia (VAP) and healthcare-associated pneumonia (HAP). The most common pathogens are *Pseudomonas aeruginosa*, *Klebsiella pneumoniae*, *Acinetobacter baumannii*, *Escherichia coli* and *Enterobacter* species. These organisms have emerged as multidrug-resistant and thus limit therapeutic options and are associated with poor clinical outcomes [3].

In the severest cases of gram-negative pneumonia, mechanical ventilation is a major risk factor. Endotracheal intubation exposes the patient to the risk of lower respiratory tract infections due to three reasons: (1) the absence of the natural mechanisms of airway defense, (2) the impairment of mucociliary clearance and (3) the predisposition to microaspiration of contaminated secretions [4]. Additionally, patients who are hospitalized for extended periods in the intensive care unit or who have received prior antibiotic therapy, have underlying medical conditions, immunosuppression, and have undergone invasive medical procedures are at an increased risk of being infected with resistant gram-negative organisms [5]. Complications in patients with gram-negative pneumonia who require mechanical ventilation are significant, such as septic shock, acute respiratory distress syndrome, multiorgan dysfunction, longer duration of mechanical ventilation, longer length of hospital and intensive care unit (ICU) stay, and higher healthcare costs. There is a high mortality risk when patients are infected with multidrug-resistant or extensively drug-resistant pathogens; therefore, prompt identification and proper antimicrobial treatment assume critical importance [6].

A few studies have identified potential risk factors for mortality in patients with severe pneumonia who are mechanically ventilated, such as advanced age, high severity scores, septic shock, delayed administration of appropriate antibiotic therapy, renal dysfunction, hypoalbuminemia, prolonged mechanical ventilation, and a diagnosis of multidrug-resistant organism [7]. But the relative importance of these factors is different in different populations and in various health care settings. In 2021, there were an estimated 2.2 million deaths due to lower respiratory infections worldwide, and they were among the top 10 causes of death globally [8]. Additionally, in low- and middle-income countries (LMICs), the burden is exacerbated due to poor access to diagnostic services, poor antimicrobial stewardship, resource limitations, and high frequency of antimicrobial resistance. In Pakistan, gram-negative microorganisms constitute a major proportion of nosocomial infections, and *A. baumannii*, *K. pneumoniae*, and *P. aeruginosa* are reported to be multidrug-resistant [9]. Gram-negative pneumonia epidemiology has changed significantly in the last 20 years. Multidrug-resistant (MDR), extensively drug-resistant (XDR), and carbapenem-resistant organisms have made treatment more difficult and challenging [10]. For patients who are most ill, carbapenem-resistant *A. baumannii*, extended-spectrum β-lactamase (ESBL)-producing *K. pneumoniae*, and multidrug-resistant *P. aeruginosa* are becoming more prevalent and are linked to delayed initiation of appropriate therapy, longer hospital stays, and poor outcomes. Carbapenem-resistant *A. baumannii*, *P. aeruginosa*, and Enterobacterales (CRE) are priority pathogens for which new antimicrobial drugs are needed, according to the World Health Organization [11,12].

Objective

The objective of this study is to determine the in-hospital mortality rate and identify predictors of mortality among intubated and mechanically ventilated adults with microbiologically confirmed gram-negative pneumonia treated at a tertiary-care hospital in Wah Cantonment, Pakistan, with particular emphasis on locally prevalent pathogens, antimicrobial resistance patterns, and appropriateness of empirical antibiotic therapy.

## Materials and methods

This retrospective cohort study was conducted at the Department of Medicine, Pakistan Ordnance Factory (POF) Hospital, Wah Medical College, Wah Cantt, Pakistan, from May 2025 to November 2025. The overall number of adult patients with gram-negative pneumonia who were endotracheally intubated and required invasive mechanical ventilation was 178. The sampling technique was non-probability consecutive sampling, which was designed to include all eligible patients in the study. Patients 18 years of age or older who were diagnosed with gram-negative pneumonia based on positive respiratory culture (endotracheal aspirate, bronchoalveolar lavage, or sputum) and had radiographic evidence of pneumonia, who were endotracheally intubated and on invasive mechanical ventilation for 48 hours or more were included in the study. Patients with polymicrobial pneumonia (fungal or viral) and insufficient medical history, who had previous tracheostomy, who underwent mechanical ventilation for reasons other than pneumonia, who were immunocompromised (active malignancy, chemotherapy), who had HIV infection with CD4 counts <200 cells/mm³, who had solid organ transplantation, and who were transferred to other facilities before hospital outcome could be determined were excluded.

Data collection

The medical records of patients who met the inclusion criteria for the study were then reviewed retrospectively after obtaining approval from the Institutional Review Board. Demographic data such as age, gender, BMI, smoking history, diabetes mellitus, chronic obstructive pulmonary disease (COPD), chronic kidney disease, cardiovascular disease, and malignancies were documented. Clinical data collected were source of admission, type of pneumonia, duration of symptoms, need for vasopressors, severity scores on admission to the ICU (Acute Physiology and Chronic Health Evaluation II (APACHE II) score [13] and Sequential Organ Failure Assessment (SOFA) score) [14], septic shock, mechanical ventilation, length of stay in the ICU, and length of hospital stay. Laboratory databases were used to obtain microbiological data, such as the presence of gram-negative organisms and their sensitivity to antimicrobial agents. Empirical antibiotic therapy, appropriateness of initial antimicrobial therapy, and development of multidrug-resistant infection were also recorded. Laboratory parameters were measured at the time of admission to the ICU, such as complete blood count, blood urea nitrogen levels, blood creatinine levels, serum albumin levels, levels of C-reactive protein, procalcitonin, arterial blood gas concentrations, and serum lactate concentrations. The main outcome was in-hospital mortality. Secondary outcomes were mechanical ventilation, length of stay in the ICU, hospital length of stay, septic shock, and acute kidney injury.

Data analysis

Data were keyed and analyzed using SPSS 26.0 (IBM Corp, Armonk, NY). Numerical data were reported as mean±SD or median (interquartile range) depending on the distribution of data, and categorical variables reported as frequencies with percentages. Independent samples t-test or Mann-Whitney U test were used for continuous variables, and chi-square test or Fisher's exact test were used for categorical variables to compare survivors with non-survivors. Variables that had a p-value <0.20 from univariate analysis were put into a multivariable logistic regression model to determine independent predictors of in-hospital mortality. A p-value of ≤0.05 was considered statistically significant.

## Results

A total of 178 intubated and mechanically ventilated adults with gram-negative pneumonia were included. The mean age was 61.8±15.2 years, and 112 (62.9%) were men. Common comorbidities included hypertension in 94 patients (52.8%), diabetes mellitus in 81 patients (45.5%), COPD in 51 patients (28.7%), cardiovascular disease in 48 patients (27.0%), and chronic kidney disease in 39 patients (21.9%). Septic shock at admission was present in 72 (40.4%) patients. The mean APACHE II and SOFA scores were 23.8±7.1 and 9.6±3.4, respectively, while the mean duration of mechanical ventilation was 11.7±6.8 days (Table 1).

**Table 1:** Baseline Demographic and Clinical Characteristics of the Study Population APACHE II score: Acute Physiology and Chronic Health Evaluation II; SOFA: Sequential Organ Failure Assessment.

Variable	Total (n=178)
Age, years	61.8±15.2
Male sex	112 (62.9%)
BMI, kg/m²	27.1±5.4
Current smoker	68 (38.2%)
Diabetes mellitus	81 (45.5%)
Hypertension	94 (52.8%)
Chronic kidney disease	39 (21.9%)
Chronic obstructive pulmonary disease	51 (28.7%)
Cardiovascular disease	48 (27.0%)
Septic shock at admission	72 (40.4%)
APACHE II score	23.8±7.1
SOFA score	9.6±3.4
Serum albumin, g/dL	2.8±0.7
Lactate, mmol/L	3.2±1.8
Duration of mechanical ventilation, days	11.7±6.8
ICU stay, days	14.5±8.2

The most commonly isolated gram-negative organism was *A. baumannii* in 58 (32.6%) patients, followed by *K. pneumoniae* in 47 (26.4%), *P. aeruginosa* in 35 (19.7%), *E. coli* in 21 (11.8%), and *Enterobacter* species in 17 (9.6%). Multidrug-resistant organisms were identified in 93 (52.2%) patients, while carbapenem-resistant isolates were present in 67 (37.6%). Empirical antibiotics were appropriate in 119 (66.9%) patients and inappropriate in 59 patients (33.1%) (Table 2).

**Table 2:** Microbiological Profile and Antimicrobial Resistance Patterns Data are presented for 178 patients with microbiologically confirmed gram-negative pneumonia. A total of 178 gram-negative bacterial isolates were recovered, with one isolate analyzed per patient. Frequencies and percentages of bacterial isolates and antimicrobial resistance patterns are reported.

Variable	Frequency, n (%)
Acinetobacter baumannii	58 (32.6%)
Klebsiella pneumoniae	47 (26.4%)
Pseudomonas aeruginosa	35 (19.7%)
Escherichia coli	21 (11.8%)
*Enterobacter* species	17 (9.6%)
Multidrug-resistant organism	93 (52.2%)
Carbapenem-resistant isolate	67 (37.6%)
Appropriate empirical antibiotics	119 (66.9%)
Inappropriate empirical antibiotics	59 (33.1%)

Non-survivors were significantly older than survivors (67.8±14.2 vs. 57.3±14.1 years; p<0.001) and had higher APACHE II scores (28.7±6.4 vs. 20.2±5.8; p<0.001) and SOFA scores (12.0±3.1 vs. 7.8±2.7; p<0.001). Septic shock was more frequent among non-survivors (48 (63.2%) vs. 24 (23.5%); p<0.001), as were multidrug-resistant infections (51 (67.1%) vs. 42 (41.2%); p=0.001) and inappropriate empirical antibiotics (36 (47.4%) vs. 23 (22.5%); p=0.001) (Table 3).

**Table 3:** Comparison of Survivors and Non-Survivors Data are presented as mean±SD or n (%). Independent samples t-test was used for continuous variables, while chi-square test was used for categorical variables. Both APACHE II and SOFA scores were significantly higher among non-survivors than survivors. The mean APACHE II score was 28.7±6.4 among non-survivors compared with 20.2±5.8 among survivors (t=9.12, p<0.001). Similarly, the mean SOFA score was 12.0±3.1 in non-survivors and 7.8±2.7 in survivors (t=9.44, p<0.001). APACHE II score: Acute Physiology and Chronic Health Evaluation II; SOFA: Sequential Organ Failure Assessment.

Variable	Survivors (n=102)	Non-survivors (n=76)	Test statistic	p-value
Age, years	57.3±14.1	67.8±14.2	t=4.89	<0.001
Male sex	61 (59.8%)	51 (67.1%)	χ²=1.00	0.32
APACHE II score	20.2±5.8	28.7±6.4	t=9.12	<0.001
SOFA score	7.8±2.7	12.0±3.1	t=9.44	<0.001
Septic shock	24 (23.5%)	48 (63.2%)	χ²=28.39	<0.001
Multidrug-resistant infection	42 (41.2%)	51 (67.1%)	χ²=11.74	0.001
Inappropriate empirical antibiotics	23 (22.5%)	36 (47.4%)	χ²=12.11	0.001
Serum albumin, g/dL	3.0±0.6	2.5±0.6	t=5.50	<0.001
Lactate, mmol/L	2.6±1.4	4.1±1.9	t=5.81	<0.001
Mechanical ventilation duration, days	10.2±5.7	13.8±7.5	t=3.50	<0.001

Multivariable logistic regression identified several independent predictors of in-hospital mortality. The strongest predictor was APACHE II score ≥25 (adjusted odds ratio (OR): 3.67; 95% CI: 1.82-7.38; p<0.001), followed by septic shock (adjusted OR: 2.94; 95% CI: 1.41-6.14; p=0.004), inappropriate empirical antibiotics (adjusted OR: 2.78; 95% CI: 1.35-5.72; p=0.006), serum albumin <2.5 g/dL (adjusted OR: 2.56; 95% CI: 1.24-5.28; p=0.011), multidrug-resistant infection (adjusted OR: 2.31; 95% CI: 1.16-4.59; p=0.017), age ≥65 years (adjusted OR: 2.18; 95% CI: 1.12-4.24; p=0.022), and lactate >2 mmol/L (adjusted OR: 2.12; 95% CI: 1.03-4.38; p=0.041) (Table 4).

**Table 4:** Multivariable Logistic Regression Analysis of Predictors of In-Hospital Mortality Multivariable logistic regression was performed to identify independent predictors of in-hospital mortality. Variables with p <0.20 in the univariate analysis were entered into the model. Model statistics: −2 Log Likelihood=176.4; Nagelkerke R²=0.48; Hosmer-Lemeshow goodness-of-fit test χ²=6.72, df=8, p=0.567; Overall model χ²=82.31, df=7, p<0.001. Reference categories are shown for each predictor. APACHE II score: Acute Physiology and Chronic Health Evaluation II; SOFA: Sequential Organ Failure Assessment.

Predictor	Reference Category	Adjusted OR	95% CI	Wald χ²	p-value
Age ≥65 years	Age <65 years	2.18	1.12-4.24	5.27	0.022
APACHE II score ≥25	APACHE II score <25	3.67	1.82-7.38	13.54	<0.001
Septic shock	No septic shock	2.94	1.41-6.14	8.18	0.004
Multidrug-resistant infection	No multidrug-resistant infection	2.31	1.16-4.59	5.69	0.017
Inappropriate empirical antibiotic therapy	Appropriate empirical antibiotic therapy	2.78	1.35-5.72	7.58	0.006
Serum albumin <2.5 g/dL	Serum albumin ≥2.5 g/dL	2.56	1.24-5.28	6.49	0.011
Lactate >2 mmol/L	Lactate ≤2 mmol/L	2.12	1.03-4.38	4.17	0.041

The overall in-hospital mortality rate was 76 (42.7%), while ICU mortality was 68 (38.2%). Acute kidney injury developed in 59 (33.1%) patients, and 24 (13.5%) required renal replacement therapy (Table 5).

**Table 5:** Clinical Outcomes of Mechanically Ventilated Patients

Outcome	Total (n=178)
In-hospital mortality	76 (42.7%)
ICU mortality	68 (38.2%)
Acute kidney injury	59 (33.1%)
Requirement for renal replacement therapy	24 (13.5%)
Tracheostomy performed	31 (17.4%)
Ventilator-associated complications	44 (24.7%)
ICU length of stay, days	14.5 ± 8.2
Total hospital stay, days	19.8 ± 10.6

## Discussion

This study aimed to assess in-hospital mortality rate and its factors associated with gram-negative pneumonia in adult patients who were intubated and mechanically ventilated. The in-hospital mortality rate was high, at 42.7%, indicating the significant morbidity associated with serious gram-negative respiratory infections in the critically ill. This finding is similar to earlier research, which has shown a mortality rate of between 30% and 50% on mechanical ventilation with hospital-acquired and ventilator-associated pneumonia, especially due to multidrug-resistant gram-negative organisms [15]. The predominant age group of the study population was older adults, with a mean age of 61.8±15.2 years, and a higher proportion of males compared to women (62.9% to 37.1%). Comorbidities like hypertension (52.8%), diabetes mellitus (45.5%), chronic obstructive pulmonary disease (28.7%) and chronic kidney disease (21.9%) were very common and underscored the vulnerability of critically ill patients with severe pneumonia. Previously, it has been shown that being older and having multiple comorbid conditions is associated with susceptibility to developing severe infections and poor clinical outcomes [16]. A male predominance was observed in the study population, with men accounting for 112 (62.9%) of the participants. The proportion of men was also higher among non-survivors than survivors (67.1% versus 59.8%); however, this difference was not statistically significant (p=0.320). The greater representation of men may reflect differences in smoking exposure, occupational risks, prevalence of chronic cardiopulmonary disease, healthcare-seeking behaviour, or patterns of hospital referral. Nevertheless, because sex was not independently associated with mortality, the observed disparity should be interpreted as a demographic pattern rather than a direct prognostic factor. Further multicentre studies with adjustment for smoking, comorbidity burden, disease severity, and treatment-related variables are required to determine whether sex influences outcomes in mechanically ventilated patients with gram-negative pneumonia.

The most common pathogens were *A. baumannii* (32.6%) and *K. pneumoniae* (26.4%) and *P. aeruginosa* (19.7%). Over half of the patients (52.2%) had infections with multidrug-resistant organisms and 37.6% of the isolates were carbapenem-resistant [17]. The current results are consistent with those of previous studies conducted in high-risk settings of low- and middle-income countries that have found a high prevalence of antimicrobial resistance of gram-negative pathogens. Multidrug-resistant organisms (MDROs) are becoming more common and pose therapeutic and clinical challenges. MDROs are increasingly common and pose therapeutic and clinical challenges. Non-survivors were significantly older than survivors (67.8±14.2 vs. 57.3±14.1 years; p<0.001) and had higher disease severity scores, including APACHE II scores (28.7±6.4 vs. 20.2±5.8; p<0.001) and SOFA scores (12.0±3.1 vs. 7.8±2.7; p<0.001). These results corroborate earlier studies that showed that the severity of illness scores can be used to predict death in severe pneumonia patients who are admitted to the Intensive Care Unit (ICU) [18].

Circulatory failure is a major factor in poor outcome; septic shock occurred significantly more often in non-survivors than in survivors (63.2% vs. 23.5%, respectively; p<0.001). In the same way, non-survivors were more likely to have multidrug-resistant infections (67.1% vs. 41.2%; p=0.001) and inappropriate empirical antibiotic treatment (47.4% vs. 22.5%; p=0.001). Adequate or delayed use of appropriate antimicrobials has been demonstrated in previous studies to be associated with increased mortality in severe infections with gram-negative bacteria, reinforcing the need for early administration of appropriate antibiotics in severe gram-negative infections [19]. Decreased serum albumin and elevated serum lactate were also linked to an increase in mortality. Non-survivors had significantly lower serum albumin levels (2.5±0.6 vs. 3.0±0.6 g/dL; p<0.001) and higher lactate levels (4.1±1.9 vs. 2.6±1.4 mmol/L; p<0.001) than survivors. Increased lactate levels are associated with greater disease severity, and reduced albumin levels are associated with poor nutritional status and systemic inflammation [20].

In multivariate logistic regression analyses, age ≥65 years, APACHE II score ≥25, septic shock, multidrug-resistant infection, inappropriate empirical antibiotic therapy, serum albumin <2.5 g/dL and lactate >2 mmol/L emerged as independent risk factors for in-hospital mortality. These variables were most strongly associated with mortality with APACHE II score ≥25 showing the highest adjusted odds ratio (aOR: 3.67; 1.82-7.38; p<0.001). The results of this study indicate that the risk of mortality is strongly influenced by patient-related factors and by factors related to the treatment. Multidrug-resistant organisms and inappropriate empirical antibiotic use were prevalent in this study, highlighting the need for strong antimicrobial stewardship programs, frequent local antimicrobial surveillance, and the use of evidence-based treatment practices. Very early recognition of high-risk patients through the use of validated severity scores and markers of prognosis may help to implement timely interventions and facilitate improved outcomes. The results of this study need to be viewed with the following limitations: This is a retrospective design and may have resulted in information bias and may not have included all clinical variables. The study was carried out in one center, and thus, the results may not be generalizable. Furthermore, long-term results after leaving the hospital were not evaluated. These results need to be confirmed in multicenter prospective studies, and strategies to decrease mortality in mechanically ventilated patients with gram-negative pneumonia should be investigated.

## Conclusions

It is concluded that mechanically ventilated adults with microbiologically confirmed gram-negative pneumonia treated at this tertiary-care centre in Pakistan experienced a high in-hospital mortality rate of 42.7%, which was broadly comparable to the 30%-50% mortality reported in previous studies of severe hospital-acquired and ventilator-associated pneumonia. The associations of mortality with advanced age, higher APACHE II and SOFA scores, septic shock, hypoalbuminaemia, elevated lactate levels, multidrug-resistant infection, and inappropriate empirical antibiotic therapy were also consistent with established international evidence. However, the present study provided important local insights by demonstrating the predominance of *A. baumannii* and *K. pneumoniae*, together with particularly high frequencies of multidrug resistance and carbapenem resistance. APACHE II score ≥25 was the strongest independent predictor of mortality, while inappropriate empirical antibiotic therapy represented an important potentially modifiable risk factor.
